# Association of limbic system-associated membrane protein (LSAMP) to male completed suicide

**DOI:** 10.1186/1471-2350-9-34

**Published:** 2008-04-23

**Authors:** Anne Must, Gunnar Tasa, Aavo Lang, Eero Vasar, Sulev Kõks, Eduard Maron, Marika Väli

**Affiliations:** 1 Institute of Physiology, Tartu University, Ravila 19, Tartu 50411, Estonia; 2 Institute of General and Molecular Pathology, Tartu University, Ravila 19, Tartu 50411, Estonia; 3 Psychiatry Clinic of Tartu University Hospital, Raja 31, Tartu 50417, Estonia; 4 Institute of Pathological Anatomy and Forensic Medicine, Tartu University, Ravila 19, Tartu 50411, Estonia

## Abstract

**Background:**

Neuroimaging studies have demonstrated volumetric abnormalities in limbic structures of suicide victims. The morphological changes might be caused by some inherited neurodevelopmental defect, such as failure to form proper axonal connections due to genetically determined dysfunction of neurite guidance molecules. Limbic system-associated membrane protein (LSAMP) is a neuronal adhesive molecule, preferentially expressed in developing limbic system neuronal dendrites and somata. Some evidence for the association between LSAMP gene and behavior has come from both animal as well as human studies but further investigation is required. In current study, polymorphic loci in human LSAMP gene were examined in order to reveal any associations between genetic variation in *LSAMP *and suicidal behaviour.

**Methods:**

DNA was obtained from 288 male suicide victims and 327 healthy male volunteers. Thirty SNPs from LSAMP gene and adjacent region were selected by Tagger algorithm implemented in Haploview 3.32. Genotyping was performed using the SNPlex™ (Applied Biosystems) platform. Data was analyzed by Genemapper 3.7, Haploview 3.32 and SPSS 13.0.

**Results:**

Chi square test revealed four allelic variants (rs2918215, rs2918213, rs9874470 and rs4821129) located in the intronic region of the gene to be associated with suicide, major alleles being overrepresented in suicide group. However, the associations did not survive multiple correction test. Defining the haplotype blocks using confidence interval algorithm implemented in Haploview 3.32, we failed to detect any associated haplotypes.

**Conclusion:**

Despite a considerable amount of investigation on the nature of suicidal behaviour, its aetiology and pathogenesis remain unknown. This study examined the variability in LSAMP gene in relation to completed suicide. Our results indicate that LSAMP might play a role in pathoaetiology of suicidal behaviour but further studies are needed to understand its exact contribution.

## Background

Suicide is one of the leading causes of death in Estonia across all age groups, especially among working age men [1]. Being as high as 73.2/100 000 in 1996, the annual male suicide rate has reduced to 30.7/100 000 in 2006, still being twice as high as the European average [2].

More than 90% of all cases of suicide are associated with a mental disorder, most often mood disorders, substance dependence or schizophrenia [3,4]. However, not all psychiatric patients suffering from depression or schizophrenia have suicidal ideation. Therefore, a stress-diathesis model for suicidal behaviour has been proposed, where stressors include elements like an acute psychiatric disorder and life events, and the diathesis includes biologically determined risk factors for suicidal behaviour such as personality traits of aggression/impulsivity and a heightened susceptibility to environmental stressors [5].

Neurobiological factors contributing to violence, impulsivity and anxiety in humans remain poorly understood. However, a majority of studies suggests a functional deficiency in the neural systems for emotional regulation and memory as possible substrate for the observed gene-environment interaction [6-9].

The limbic system is an integrated brain area involved in the regulation of reward, motivation, emotional expression and memory, as well as decision-making and predicting outcomes of one's behaviour. Neuroimaging studies of limbic system have demonstrated the volumetric abnormalities in these structures of depressive, schizophrenic as well as suicidal patients [10-14]. These findings have been explained with hypothalamic-pituitary-adrenal axis hyperactivity, resulting in glucocorticoid neurotoxicity [15]. It can be also proposed that the observed changes in volume of structures do not result from lifetime stress but are inborn, caused by some inherited neurodevelopmental defect.

Proper development of the nervous system includes formation of specific connections between neuronal populations. The process depends on growth cone guidance molecules that lead to correct axon targeting.

Limbic system-associated membrane protein (LSAMP) is a highly conserved adhesion molecule expressed primarily by cortical and subcortical neurons of the limbic system at early developmental stages [16-18]. It exerts dual effects, attracting limbic thalamic axons and at the same time impeding nonlimbic thalamic axons from innervating inappropriate cortical regions [19].

Animal studies have revealed that rats with lower exploratory activity have an increased expression of *Lsamp *gene in their limbic structures [20]. On the contrary, *Lsamp *knockout mice demonstrate behavioural hyperactivation in novel environments which is suggested to reflect a behavioural disinhibition in stress situations rather than diminished anxiety [21]. Up to now, there has not been many published studies about *LSAMP *and human behaviour, although we have found some evidence about *LSAMP *polymorphisms being associated with panic disorder [22,23].

Proposing that genetic variations in *LSAMP *gene might be associated with impaired development of limbic structures and therefore causing neural dysfunction that leads to suicidal behavior, the purpose of current study was to find any associations between single-nucleotide polymorphisms (SNP) in LSAMP gene and male completed suicide.

## Methods

### Subjects

Two groups of male subjects were investigated in this study: suicide victims (n = 288, mean age 42.8 years, SD = 13.69), and healthy volunteers without any history of psychiatric illness or suicide attempts (n = 327, mean age 40.5 years, SD = 14.49).

The definition of suicide was based on the results of medicolegal examination by Estonian Forensic Examination Bureau. Subjects committed suicide as follows: hanging (88%), shooting (10%), drowning (0.5%), poisoning (0.5%), penetrating lesion (0.5%), blunt lesion (0.5%). Diagnostic information about occurrence of psychopathologies prior to death was available only for a very small proportion of suicide subjects, thus this data is not presented in the current paper.

Healthy controls were recruited by newspaper advertisement in Tartu, Estonia. They were interviewed at the Clinic of Psychiatry of Tartu University Clinics, using Mini International Neuropsychiatric Interview (M.I.N.I. 5.0.0). The exclusion criteria included personal or family history of psychiatric disorders, diagnosis of alcohol dependence, use of psychotropic medication and chronic bodily diseases.

For DNA extraction and further genotyping, kidney biopsies from suicide completers were collected by Estonian Forensic Examination Bureau, and venous blood from healthy subjects was collected by Psychiatry Clinic of Tartu University Hospital.

Informed consent was obtained from healthy subjects and relatives of suicide completers. The study was approved by Ethics Review Committee on Human Research, Tartu University.

### Marker selection and genotyping

Human *LSAMP *is a 635 kb gene located on 3q13.2-q21, containing seven exons.

Thirty tag SNPs were selected from *LSAMP *gene and flanking regions (150 kb fragment from 116931222–117081732; and 242 kb fragment from 117428563–117670401) using the Tagger algorithm (r^2 ^= 0.8, minor allele frequency >0.05) implemented in Haploview 3.32 [24].

DNA was obtained from tissue or blood samples by standard phenol extraction method. Loci were genotyped by SNPlex™ (Applied Biosystems) platform.

### Statistics

The genotypes were assigned by GeneMapper 3.7 (Applied Biosystems).

Haplotype blocks were delineated using the confidence interval method of Gabriel [25] implemented in Haploview 3.32. Comparison of allele/haplotype frequencies between cases and controls was done by chi square test. Permutation tests with 10 000 permutations as well as test of conformity of genotype frequencies to Hardy-Weinberg equilibrium were performed by Haploview 3.32. The demographic data calculations were made by SPSS 13.0.

## Results

### Association of single markers

All the genotyped SNPs were in Hardy-Weinberg equilibrium (p > 0.001). Comparison of allele frequencies between cases and controls using chi square test revealed significant differences for four SNPs: rs4831129, rs9874470, rs2918213 and rs2918215, as seen in Table 1. The odds ratios for associated loci were respectively 1.3 (CI: 1.01-1.67), 1.3 (CI: 1.01-1.67), 1.3 (CI: 1.00-1.62) and 1.5 (CI: 1.01-2.29).

**Table 1:** Association of single markers in *LSAMP *gene with completed suicide.

**Name**	**Pos 5'**	**Area**	**Alleles (major/minor)**	MAF* case	MAF* control	**Chi square**	P value**	**HWE p value**
rs16824996	-23333	5' flanking region	T/C	0.86	0.86	0.06	0.814	1.000
rs6787168	-20108	5' flanking region	T/C	0.72	0.7	1.06	0.303	0.443
rs4831140	-8362	5' flanking region	A/T	0.83	0.81	0.83	0.363	0.766
rs4831097	14146	intron 1	C/T	0.89	0.88	1.03	0.309	0.927
rs9289051	46772	intron 1	G/T	0.9	0.87	3.63	0.057	1.000
rs17647013	65337	intron 1	C/T	0.9	0.9	0.05	0.825	0.487
rs988803	78700	intron 1	T/A	0.8	0.79	0.47	0.494	0.200
rs7634137	94215	intron 1	T/C	0.83	0.8	1.19	0.276	0.165
rs1920191	121973	intron 1	G/A	0.75	0.73	0.58	0.446	0.260
rs2100807	140388	intron 1	A/G	0.84	0.82	0.84	0.361	0.601
rs1461131	163706	intron 1	G/A	0.61	0.63	0.38	0.538	0.939
rs16824691	181606	intron 1	T/A	0.8	0.78	0.59	0.442	0.027
**rs4831129**	**182935**	**intron 1**	**T/G**	**0.74**	**0.68**	**4.19**	**0.041**	**0.180**
rs4831089	192350	intron 1	G/A	0.61	0.64	1.24	0.266	0.774
**rs9874470**	**198730**	**intron 1**	**T/C**	**0.74**	**0.68**	**4.13**	**0.042**	**0.078**
rs6438308	209904	intron 1	G/C	0.81	0.78	1.45	0.228	0.036
rs4831124	218505	intron 1	T/C	0.66	0.63	1.59	0.208	0.551
rs2944425	565336	intron 3	C/T	0.61	0.57	1.66	0.197	0.997
rs9830559	568061	intron 3	T/C	0.6	0.62	0.76	0.384	0.578
rs1464140	569000	intron 3	T/C	0.82	0.81	0.05	0.821	0.845
rs10511350	573847	intron 3	G/C	0.91	0.9	0.49	0.485	0.757
rs6763835	590546	intron 4	C/T	0.64	0.61	0.9	0.344	0.652
rs4416377	599008	intron 4	T/C	0.79	0.78	0.6	0.437	0.010
**rs2918213**	**611939**	**intron 6**	**C/T**	**0.68**	**0.63**	**3.98**	**0.046**	**0.287**
**rs2918215**	**616902**	**intron 6**	**G/A**	**0.93**	**0.89**	**4.1**	**0.043**	**0.385**
rs2918217	617998	intron 6	G/A	0.87	0.86	0.33	0.565	0.423
rs9822311	625727	intron 6	G/C	0.71	0.68	1.06	0.303	0.020
rs2289271	635618	3' flanking region	G/A	0.59	0.54	3.21	0.073	0.801
rs2918239	682816	3' flanking region	T/G	0.85	0.84	0.46	0.498	0.787
rs2918206	715846	3' flanking region	C/T	0.59	0.56	1.16	0.282	0.054

* MAF = major allele frequency

** not corrected for multiple testing

In case of all initially associated SNPs, the major allele was observed more frequently in suicide victims than in control subjects. Comparing the predisposing allele homozygotes against other genotypes, the observed differences between cases and controls were more eminent. In case of rs4831129, frequency of TT genotype was significantly higher in suicide group, as opposed to GG or GT genotypes (chi square(1) = 6.624, p = 0.011; OR = 1.5, CI: 1.11-2.11). The same principle applied for rs9874470 where frequency of T-homozygotes in suicide group exceeded that of controls (chi square(1) = 5.964, p = 0.017; OR = 1.5, CI: 1.08-2.07).

However, the statistical significance disappeared after correcting for multiple comparisons.

### Association of haplotypes

**Figure 1:**
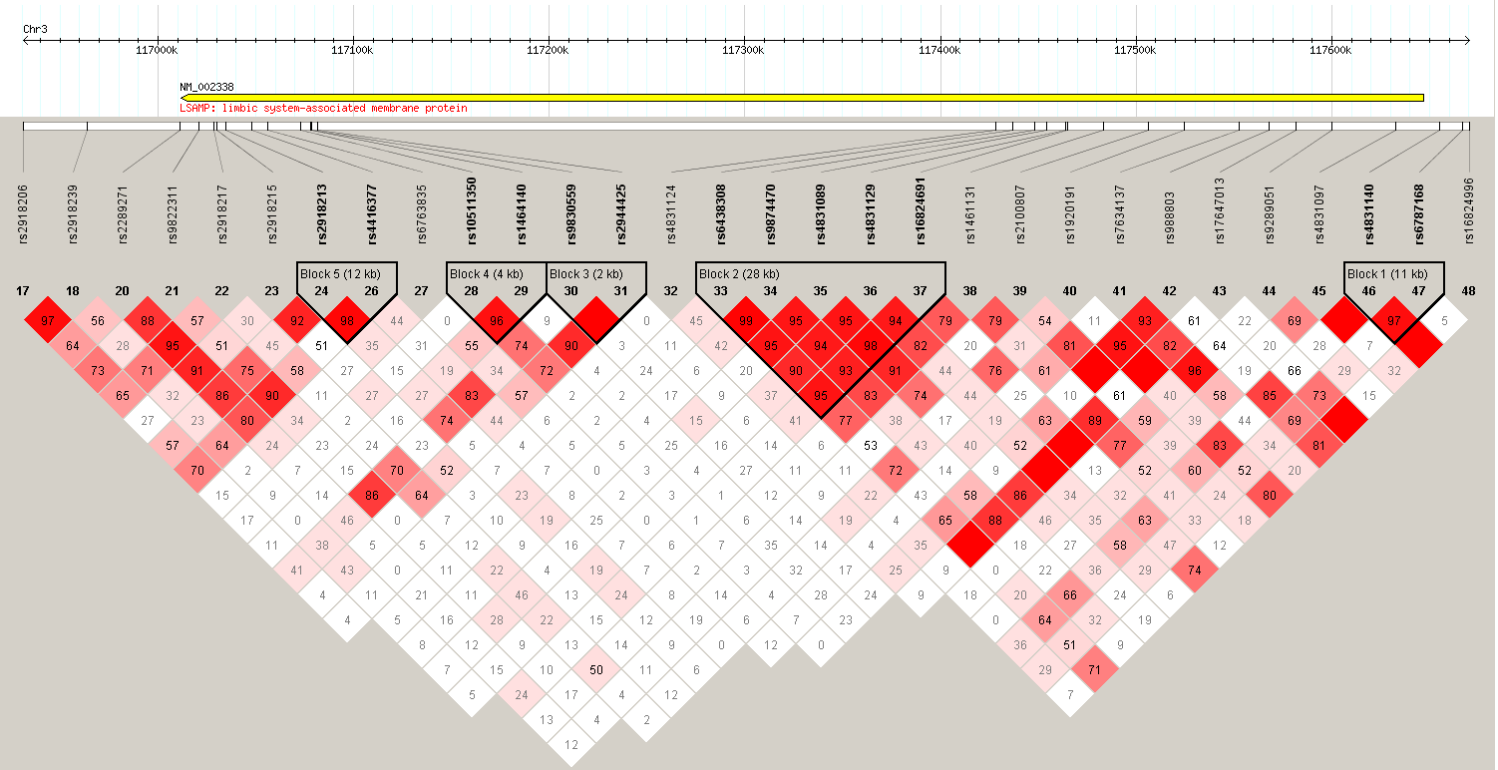
**Schematic representation of the chromosome 3 region where LSAMP gene is located with the location of the 30 tagging SNPs**. The output of LD analysis using Haploview is shown. Haplotype blocks, determined using the default confidence interval algorithm, are indicated on the LD output. Marker pairs in complete LD are indicated by an empty box.

We found five haplotype blocks across the observed chromosome fragment, based on confidence interval method (Figure 1): block 1 (rs6787168, rs4831140); block 2 (rs16824691, rs4831129, rs4831089, rs9874470, rs6438308); block 3 (rs2944425, rs9830559); block 4 (rs1464140, rs10511350) and block 5 (rs4416377, rs2918213). No statistically significant differences in haplotype block frequencies were observed between cases and controls (Table 2).

**Table 2:** Association of estimated haplotypes (Haploview) in *LSAMP *gene with completed suicide.

	**Freq case**	**Freq control**	**Chi Square**	**P Value**
Block 1*				
TA	0.72	0.69	1.08	0.3
CT	0.17	0.19	0.83	0.364
CA	0.11	0.12	0.17	0.678
Block 2*				
TTATG	0.39	0.34	2.12	0.145
TTGTG	0.34	0.32	0.29	0.593
AGGCC	0.18	0.2	1.47	0.225
TGGCG	0.07	0.1	2.84	0.092
AGGTG	0.01	0.01	0	0.985
Block 3*				
TT	0.39	0.43	1.47	0.226
CC	0.4	0.38	0.81	0.368
CT	0.2	0.19	0.15	0.697
Block 4*				
TG	0.81	0.81	0.09	0.767
CG	0.1	0.09	0.17	0.682
CC	0.09	0.1	0.54	0.463
Block 5*				
TC	0.68	0.62	3.40	0.065
CT	0.21	0.23	0.62	0.430
TT	0.11	0.15	3.31	0.069

* – haplotype combinations with less than 1% frequency are not displayed

## Discussion

In the current study we tested the hypothesis that a variation in limbic system-associated membrane protein gene is associated with completed suicide. To our knowledge, this is the first study investigating genetic variance in *LSAMP *gene associated with suicide.

Complex causative mechanisms are thought to underlie suicidal behaviour such as environmental adversities, genetic predisposition, neurotransmitter imbalance, structural and functional brain abnormalities. Highly complex interactions between all these pathways make the understanding of suicide extremely difficult.

The heritable basis of suicidal behaviour is supported by data from adoption and family studies: the concordance rate of suicide in monozygotic twins is up to 23.1%, compared to the total sample rate of 0.2% [26].

As demonstrated in numerous studies, the majority of suicide victims suffer from a psychiatric disease [3,4]. However, data from population genetic studies indicates that suicidal acts have a genetic contribution in terms of cause or diathesis that is independent of the heritability of major psychiatric disorders. This is confirmed by the simple fact that as opposed to mood disorders where female-to-male ratio is 2:1, suicide rate is four times higher among males than females [27,28].

Neuroimaging studies have demonstrated volumetric abnormalities of certain brain structures in suicide victims [13]. The structures affected belong to the limbic system, a brain structure involved in emotional processing, decision-making and predicting long-term outcomes of one's behaviour. The relatively diminished cortical modulation of lower limbic structures activity might contribute to impulsive acts and heightened susceptibility to environmental stress, the traits which are often associated with autoaggressive behaviour.

Observed changes could be applied to several causative mechanisms like glucocorticoid neurotoxicity or impaired neurodevelopment.

The effect of the LSAMP on the development of limbic circuits has gained considerable attention in the last decade. Although its expression in CNS structures was demonstrated more than 20 years ago [29], its molecular characteristics as well as specific mode of action are currently being revealed [17-19,30-33]. The research data thus far has proposed that LSAMP is a neuronal surface glycoprotein found in cortical and subcortical regions of the limbic system. During development of the limbic system, it acts as a selective homophilic adhesion molecule, facilitating formation of functional circuits between populations of limbic neurons. However, unpublished data from Philips and colleagues indicates that in mouse brain, pattern of LSAMP expression is not limited to limbic structures but appears widely all over the neocortex, particularly in sensory areas.

Little is known about the role of LSAMP in adult organism. A recent study by Catania et al shows that *Lsamp *knockout mice exhibit a maladaptive response to novel environmental stressors, expressed as behavioural disinhibition [21]. On the contrary, is has been demonstrated that high anxiety rats, as indicated by lower exploratory activity in elevated plus-maze model, show upregulation of *Lsamp *gene in amygdala and periaqueductal gray [20,34]. Very preliminary observation data from *Lsamp *knockout mice study by Philips and colleagues show that these animals exhibit mild alterations in social interaction such as lack of whisker trimming. Earlier studies indicate that barbering of cage mates is an essential form of dominant behaviour in rodents and lack of it may represent a failure to establish a normal hierarchical social interaction between animals.

According to the authors' knowledge, no studies on association between LSAMP and human behaviour have been published up to now. This is the first attempt to find an association between genetic variation in *LSAMP *gene and suicide in males. Chi square test revealed four SNPs of 30 to be associated with suicide. In all cases, the major allele of polymorphisms was overrepresented in suicide group. Moreover, two SNPs seemed to increase the risk in a dose-dependent way – the difference between study groups were more eminent if only predisposing allele homozygote frequencies were compared. However, all the associations disappeared after applying correction for multiple comparisons.

Despite none of the initial associations survived multiple testing, the results of the present study can be taken as a hint to a novel factor in suicide ethiology. However, further studies are required to see if our initial associations occurred by chance.

The potential functional consequences of the SNPs under study remain unknown at the moment. All four of the initially associated SNPs lie within the intron region, so no direct change in amino acid sequence results in the polymorphism. However, considering their conserved nature, it is possible that they are linked to a nearby functional polymorphism or act as regulatory elements.

## Conclusion

According to the results of the current study, there might be a chance that variations in LSAMP gene play a role in pathoaetiology of suicidal behaviour. However, further studies are required to reveal the nature of this finding.

## Competing interests

The authors declare that they have no competing interests.

## Authors' contributions

AM participated in design of the study, performed the genotyping and statistical analysis and drafted the manuscript. MV coordinated collection of the autopsy samples of suicide victims. EM coordinated collection of the blood samples of healthy volunteers and performed their psychiatric testing. GT coordinated the purification of DNA and helped to revise the draft. EV, SK and AL participated in design of the study, its coordination and revising the draft critically. All authors have read and approved the final manuscript.

## Pre-publication history

The pre-publication history for this paper can be accessed here:


